# On-chip-based electrochemical biosensor for the sensitive and label-free detection of *Cryptosporidium*

**DOI:** 10.1038/s41598-022-10765-0

**Published:** 2022-04-28

**Authors:** George S. Luka, Homayoun Najjaran, Mina Hoorfar

**Affiliations:** School of Engineering, University of British Columbia, 333 University Way, Kelowna, BC V1V1V7 Canada

**Keywords:** Electrochemistry, Sensors and probes

## Abstract

*Cryptosporidium*, an intestinal protozoan pathogen, is one of the leading causes of death in children and diarrhea in healthy adults. Detection of *Cryptosporidium* has become a high priority to prevent potential outbreaks. In this paper, a simple, easy to fabricate, and cost-effective on-chip-based electrochemical biosensor has been developed for the sensitive and label-free detection of *Cryptosporidium* oocysts in water samples. The sensor was fabricated using standard lithography using a mask with a 3-electrode design and modified by self-assembling a hybrid of a thiolated protein/G and the specific anti-*Cryptosporidium* monoclonal antibodies (IgG3). The electrochemical impedance spectroscopy (EIS) was employed to quantitate *C. parvum* in the range of 0 to 300 oocysts, with a detection limit of approximately 20 oocysts/5 µL. The high sensitivity and specificity of the developed label-free electrochemical biosensor suggest that this novel platform is a significant step towards the development of fast, real-time, inexpensive and label-free sensing tool for early warning and immediate on-site detection of *C. parvum* oocysts in water samples, as compared to the traditional methods (such as PCR and microscopy). Furthermore, under optimized conditions, this label-free biosensor can be extended to detect other analytes and biomarkers for environmental and biomedical analyses.

## Introduction

*Cryptosporidium* spp. is a common intestinal protozoan parasite occurring in many animal species and humans worldwide. Recently, cryptosporidiosis has become a global public health concern^1,2^. Water contamination with *Cryptosporidium* represents a significant challenge in delivering safe drinking water and a significant threat to human health^3,4^. *Cryptosporidium* oocysts have high resistance to the most common disinfectants and can remain infective and survive outside the host for up to 16 months^2,5^. *Cryptosporidium* can cause mortality in immuno-compromised individuals, especially patients with AIDS and children, and can lead to severe gastroenteritis, cryptosporidiosis in healthy adults^6,7^. In developing countries, an estimation of 30% to 50% of childhood deaths is caused by *Cryptosporidium*^2^. In developed countries, *Cryptosporidium* represents a significant risk in the water supply. One of the major outbreaks caused by *Cryptosporidium* was in Milwaukee in 1993^8^. More than 400,000 people were infected, and over 100 deaths were reported. Furthermore, *Cryptosporidium* harms the economy in both developing and developed countries. For example, the cost of illness in the 1993 Milwaukee waterborne *Cryptosporidium* outbreak was 96 million USD^9^.

The major challenge in detecting *Cryptosporidium* oocysts' early detection (without using a pre-concentration method) is their low number in a large volume of water^1,4,7^. The existing methods for detecting *Cryptosporidium* include the Environmental Protection Agency (EPA) 1623^10^, immunoassay techniques such as Enzyme-linked immunosorbent assay (ELISA)^11^, and molecular techniques such as polymerase chain reaction (PCR)^12,13^. The EPA 1623 is often insufficient, complicated with variable and low recovery efficiencies^1,2,4^. The method requires labelling *Cryptosporidium* with an immuno-fluorescence label. After successful labelling, a fluorescence microscope is used to visualize the labelled oocysts. The steps involved in this detection method make it expensive and time-consuming. Furthermore, the EPA has a limited sensitivity due to the cross-reactivity and background noise generation^2,4^. Additionally, it needs well-trained personnel, extensive sample preparation and is not suitable for on-site detection^7^.

In the last decade, immunological methods have gained significant market acceptance for detecting pathogens in environmental samples. These techniques include western blotting, enzyme-linked immunosorbent assay (ELISA), and immunofluorescence assay. Immunological assays rely on using enzyme or fluorophore-conjugated antibodies to detect the presence of specific pathogen antigen or antibody. In positive samples, the reaction between the conjugated antibody and the antigen or antibody in question results in the formation of a stable immunocomplex, leading to a change in color or fluorescence. The intensity of the fluorescence or the color is directly proportional to the target antigen or antibody concentration. These methods are easy to perform^14^, specific^15^, sensitive^16^, reproducible^17^, and have a short turnaround time compared to the culturing technique^18^. Many immunological techniques have been developed and used to detect *Cryptosporidium* to overcome the limitations of fluorescence microscopy-based methods^19^. However, despite the high sensitivity and specificity of some of the immunological methods, they generate irreproducible data, resulting in false-positive and false-negative results^20^. They suffer from interference from contaminants in the samples such as non-targeted DNA, cells or proteins^21^. Furthermore, these techniques need advanced laboratories, bulky equipments (for visual determination and quantification), trained personnel, expensive, and unsuitable for real-time detection^22,23^.

Recently, molecular techniques such as PCR have extensively been used to detect specific *Cryptosporidium* DNA sequences to overcome the drawbacks of fluorescence microscopy and immunological methods^19,24^. However, these techniques require complex sample preparation steps, such as cell extraction followed by nucleic acid purification and amplification. These drawbacks make molecular techniques a complicated approach, as they require expensive lab-based instruments and highly trained personnel^25^. Furthermore, these techniques have not yet been widely used for on-site detection nor adopted in commercial diagnostic laboratories^2^. Therefore, the development of label-free, portable, flexible, rapid and reliable sensing platforms for detecting *Cryptosporidium* in water samples in a real-time manner has become of necessity. A few sensing methods have been proposed to detect *Cryptosporidium*; however, for better sensitivity and selectivity, these sensors require selective biological recognition elements such as those implemented in biosensors^2^.

Biosensors are powerful analytical tools and can detect a wide range of applications ranging from medical diagnostics, food safety, drug discovery to security and defence^26^. A typical biosensor comprises a recognition element (such as antibodies^4^, nucleic acids^27^, enzymes^28,29^, aptamers^30^ or whole cells^31^), transducer, and detector^26^. The recognition element dictates the biosensor's specificity and selectivity, while the transducer transforms the response produced from a biorecognition event to a measurable signal. The signal is then detected using a suitable detector^26,29^. Compared to conventional lab-based techniques, biosensors are reliable, cost-effective, easy to use, and accurate due to their reusability, high specificity and selectivity, portability, and real-time response^26^. However, from the cost point of view, many biosensors must be developed based on label-free methods. Label-free detection methods also reduce the time and steps involved, including the expensive labelling protocols, which facilitate real-time detection^4^. Among different label-free methods, electrochemical-based detection such as electrochemical impedance spectroscopy (EIS) has shown many advantages, including simplicity^32^, scalability^33^, portability^34^, ease to use^35^, sensitivity^35^, and low power consumption^36^. On-chip-based biosensors have become powerful emergent tools for developing miniaturized sensing platforms for on-site detection and diagnostics. These on-chip-based sensors have the capacity of accurately detecting the analyte of interest using small volumes of samples with excellent specificity and sensitivity, reproducibility, and throughput. Thus, enabling (a) rapid detection, (b) reduced costs due to lower sample and reagents, (c) creating isolated reaction sites for multiplex analysis, and (d) reduced power consumption^26,37^.

Moreover, these sensors can be mass-produced at low‐costs, and they can be used as disposable sensing platforms to detect different pathogens in minute volumes rapidly^38^. This allows many analyses to be performed remotely outside the laboratory (at the point of need)^26^. Therefore, on-chip detection could be used as the bridging gap between detection in advanced laboratories and detection on-site, providing substantial advancements in the field of point-of-care (POC) diagnostic.

Recently, a screen-printed aptamer-based electrochemical biosensor^1^ was developed for detecting *C. parvum* oocysts in spiked fresh fruits. In this study, DNA aptamers were developed and immobilized via a thiolated ssDNA primer onto a screen-printed carbon electrode modified with gold nanoparticles. The immobilized DNA aptamer was used as the molecular recognition element. The square wave voltammetry (SWV) method was used as the detection technique. The reported detection limit of the sensor was reported as 100 oocysts/30 µL. One point to emphasize regarding this study is the cost/labor associated with developing aptamers and their commercial availability. Furthermore, sensor modification with gold nanoparticles was used to enhance the sensitivity.

This paper presents a simple, easy to fabricate, cost-effective, and scalable chip-based electrochemical biosensor for sensitive and label-free *Cryptosporidium* detection. The biosensor's sensing interface is based on forming a self-assembled monolayer of thiolated-protein/G/anti-*Cryptosporidium* antibodies (commercially available) as the capture probe onto a microfabricated gold electrode. The self‐assembled monolayer (SAM) created on the chip-based microfabricated gold (Au) electrode provided a reproducible and well‐ordered layer for immobilization anti-*Cryptosporidium* antibody. EIS was used for measuring the change in the sensor film resistance and electron charge-transfer permittivity due to the formation of the *Cryptosporidium*-antibody complex. The proposed chip-based biosensor has comparable sensitivity, efficiency, and detection limits to other biosensors reported in the literature^1^. However, the sensor developed here does not need expensive aptamers to enhance selectivity. Moreover, the sensor developed here does not require expensive substrates or complicated fabrication (such as gold nanoparticle-modified screen-printed carbon electrode1) to enhance sensitivity. Thus, the proposed on-chip sensing platform has a great potential to be used as an inexpensive, flexible, portable, and reliable sensing platform for the early detection of *Cryptosporidium* in water samples*.*

## Materials

Purified *Cryptosporidium* parvum oocysts were purchased from Waterborne, Inc., New Orleans, LA, the USA, at a concentration of 10^6^ oocysts/mL. The oocysts were kept in 0.1 M phosphate-buffered saline (PBS), pH 7.4 at 4 °C. *Cryptosporidium* oocyst-specific antibody, i.e., IgG3 subclass monoclonal mouse, was purchased from ABD Serotec (Currently Biorad, Burlington, Ontario, Canada). Protein/G/thiol was purchased from protein MOD (Madison, Wisconsin, USA). Bovine serum albumin (BSA), sodium phosphate dibasic, and sodium phosphate monobasic monohydrate were purchased from Sigma (Sigma-Aldrich, city, state, Canada). All other reagents and solvents were of analytical grade, and ultrapure water was used throughout the experiments.

## Experimental methods

### Sensor fabrication

**Figure 1:**
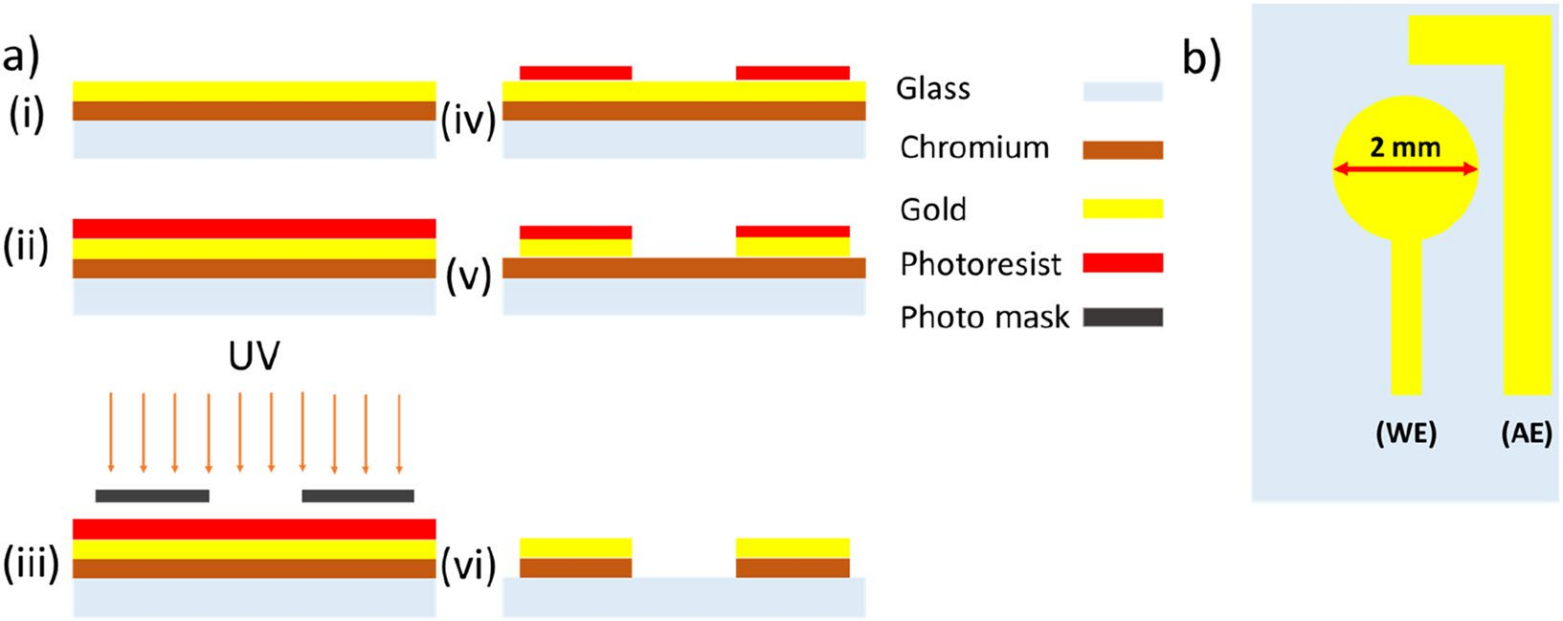
The fabricated electrochemical-based biosensor. (**a**) Fabrication steps, (**b**) Layout of the electrochemical sensing electrodes, working electrode (WE) and auxiliary electrode (AE).

The fabrication of the chip-based electrochemical immunosensor was performed using the steps shown in Fig. 1a. A piranha solution was used to clean all glass slides, followed by an oxygen plasma treatment for 10 min. Chromium and gold layers with thicknesses of 50 nm and 250 nm, respectively, were sputtered (Angstrom Engineering) in an argon atmosphere on a glass substrate. The sensing platform consisting of the working electrode (WE with 2 mm in diameter) and auxiliary electrode (AE) was patterned using the standard lithography process (Fig. 1b). An external Ag/AgCl reference electrode was used for the measurement.

### On-chip immunosensor preparation

**Figure 2:**
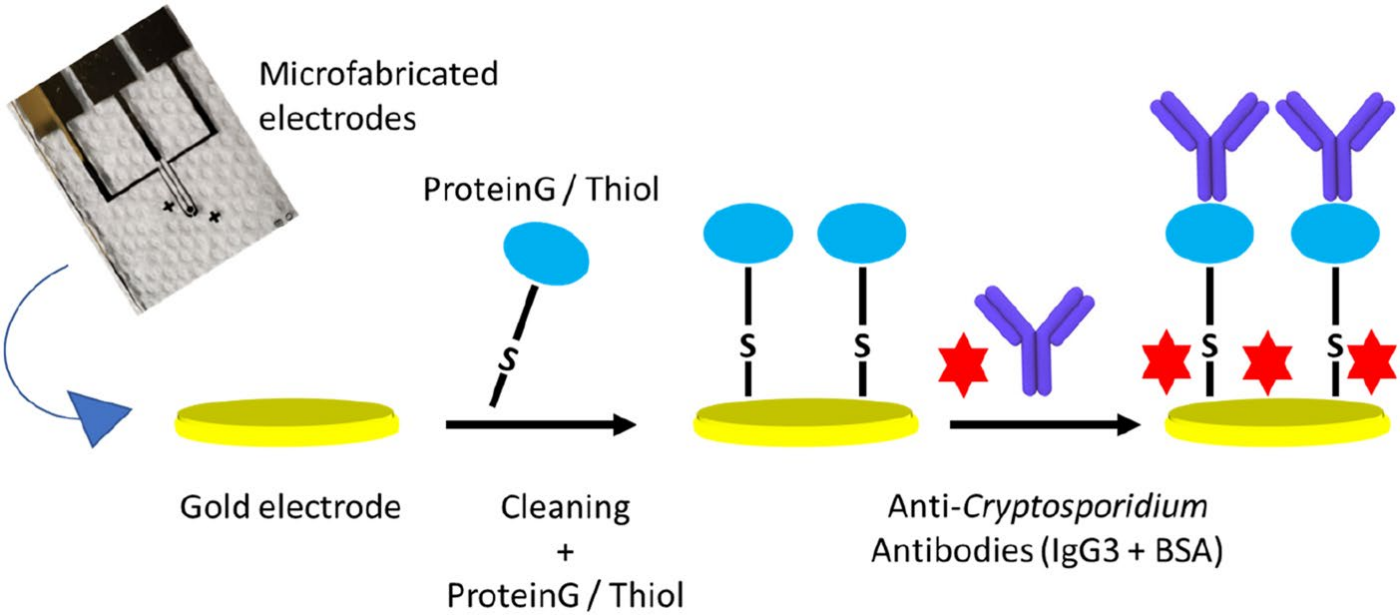
The step-wise process of coating the surface of the microfabricated Au WE with SAM and the immobilization of anti-*Cryptosporidium* antibodies onto the microfabricated Au WE. Clean Au, WE were initially functionalized by incubating it with thiolated protein/G for 48 h at 4 °C. The sensors-SAM were then incubated with 3 µL of 100 µg/mL specific anti-*Cryptosporidium* antibodies (IgG3) at 4 °C for 24 h. Lastly, the modified sensors were blocked by incubation with 100 mM BSA at 4 °C for 2 h.

Prior to surface modification, the fabricated electrodes were immersed in piranha solution of (H_2_SO_4_-H_2_O_2_ 3:1 (v/v)) for 30 s and rinsed with ultrapure water. All electrodes were subsequently cleaned electrochemically by cycling them in a 0.5 M KOH (basic) solution in the range of − 2 V to 0 V versus the reference electrode (Ag/AgCl), followed by cycling them in a 0.5 M H_2_SO_4_ (acidic) solution in the range of 0 to + 1.5 V separately. Plasma oxygen was then used to treat the fabricated gold electrodes. For the formation of SAM, the cleaned electrodes were immediately incubated with recombinant protein/G/thiol at 4 °C for 48 h (Fig. 2). The recombinant protein/G/thiol monolayer served as the linker between the Au microfabricated gold WE and the anti-*Cryptosporidium* antibodies. The modified sensing electrodes were then thoroughly rinsed with ultrapure water followed by phosphate buffer solution (PBS) (to wash away unbound recombinant Protein/G/thiol) and blown dry by N_2_ (g). The sensor/SAM was incubated with 3 µL of 100 µg/mL specific anti-*Cryptosporidium* antibodies (IgG3) at 4 °C for 24 h. The sensors/SAM-anti-*Cryptosporidium* antibodies were rinsed again with PBS (to wash away unbound antibodies) and blown dry. The modified microfabricated Au electrode surfaces were then blocked by incubation with 100 mM BSA at 4 °C for 2 h. Lastly, the electrodes were washed several times with PBS to remove excess BSA^3,4^ and blown dry. The prepared electrodes (Au‐thiolated protein/G/Abs/BSA) were stored in dry conditions at 4 °C.

### Electrochemical measurements of sam and antibody immobilization

**Figure 3:**
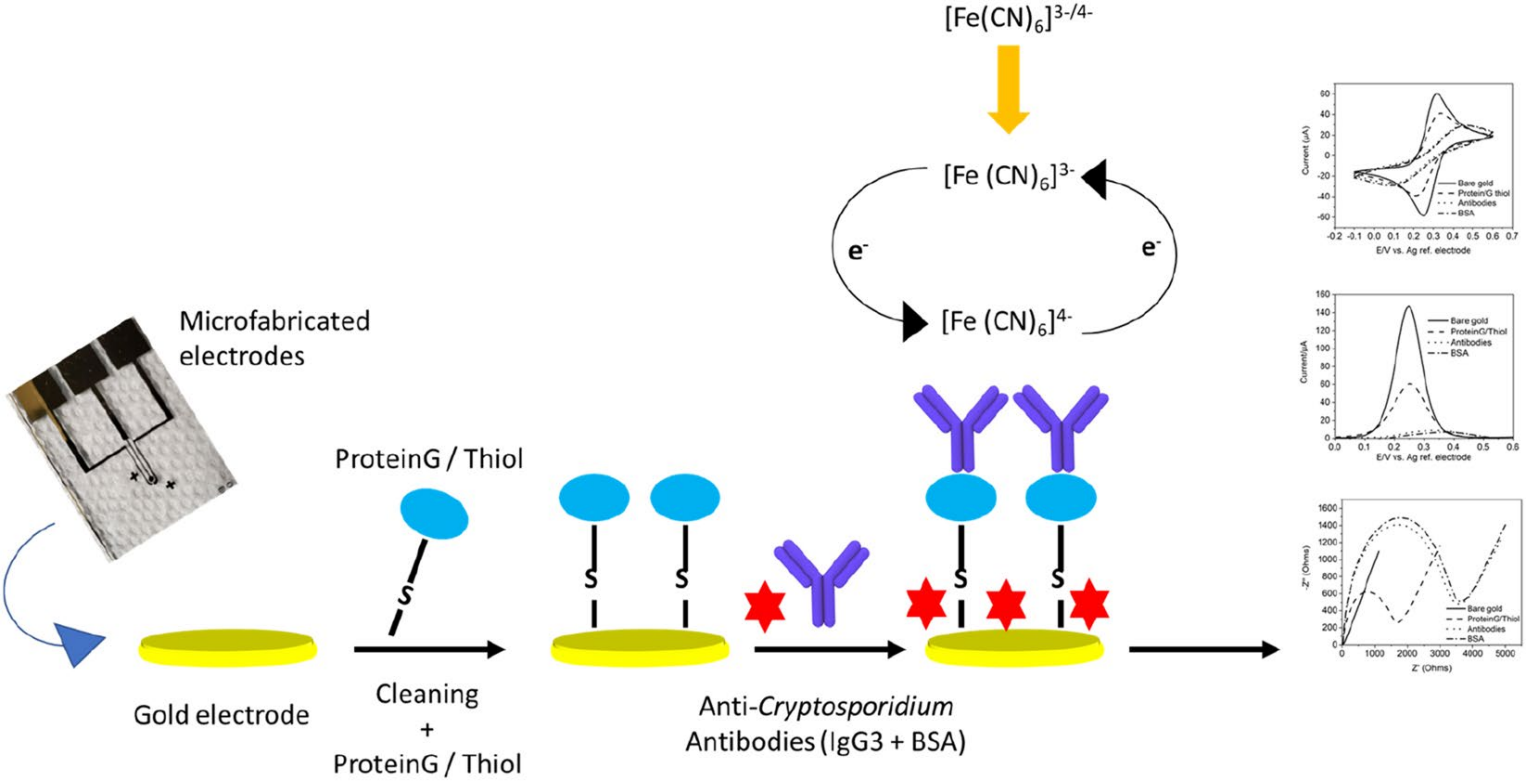
Characterizations of the modified sensor surface using cyclic voltammetry (CV), square wave voltammetry (SWV), and electrochemical impedance spectroscopy (EIS).

The modification of the on-chip fabricated electrodes with SAM and antibody immobilization was checked using different electrochemical methods (CV, SWV, and EIS). In a typical electrochemical experimental setup, the modified anti-*Cryptosporidium* antibody microfabricated Au electrode (2 mm) and AE were used as the working electrode and auxiliary electrode, respectively. An external Ag/AgCl/3.0 M KCl electrode was used as the reference electrode. A salt bridge, filled with agar and 1 M KNO_3_ aqueous solution, was used to reduce chloride ion diffusion into the electrolyte solution. Electrochemical measurements were recorded using the VersaSTAT 4 electrochemical station (Princeton Applied Research) in the bare gold, SAM, and antibodies in the three-electrode setup. All electrochemical measurements were started at the open circuit potential (OCP) and were performed in the presence of an electrolyte consisting of a 5 mM [Fe(CN)_6_]^3−/4−^ aqueous solution with 1 M NaClO_4_ (as the supporting electrolyte). To be consistent, an open circuit potential was used in all electrochemical measurements (Fig. 3).

The CV measurements started with a 30 s quiet time followed by a positive scan polarity, which was swept between the potential range of − 200 mV to + 600 mV (against Ag/AgCl) at a scan rate of 50 mV/s mV/s. The SWV measurements were conducted in the potential range of − 100 mV to 600 mV with an amplitude of 25 mV and a 2 s quiet time. The EIS measurements were performed in the frequency range of 0.1 Hz–100 kHz with a 5 mV amplitude and started with a 30 s quiet time.

### Contact angle measurements

**Figure 4:**
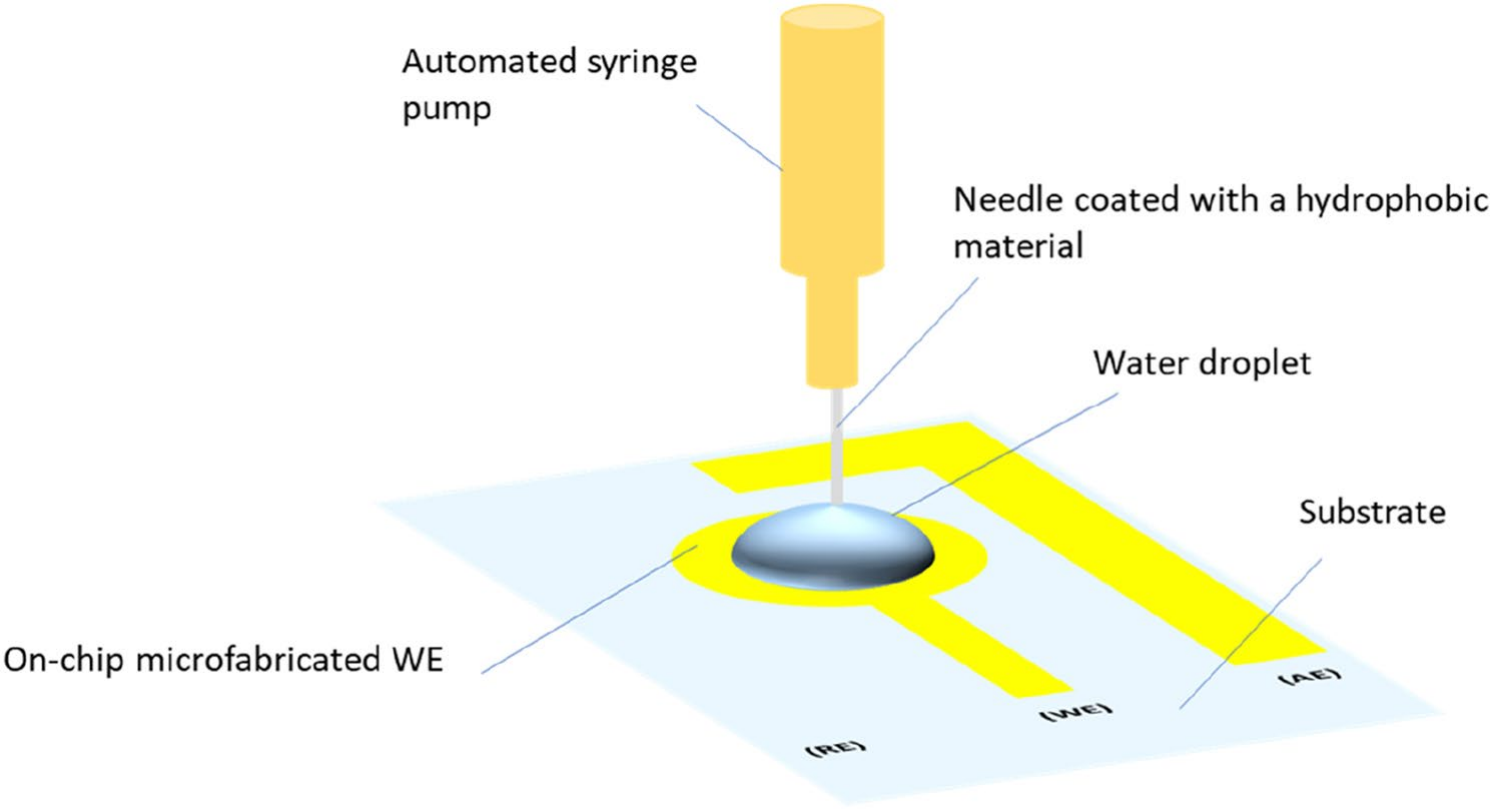
Schematic illustrating the experimental setup for contact angle measurement.

Contact angle measurements were conducted to further verify the immobilization of the antibodies onto the electrodes. In essence, the contact angle measurement was conducted (as explained previously in^3,4^ to ensure the presence of the SAM and antibodies on the surface based on the changes in the surface properties from hydrophilic to superhydrophilic (Fig. 4). The procedure involved preparing two substrates: one with chromium and gold layers (referred to as blank) and the other with the SAM and immobilized antibodies on the chromium and gold layers (referred to as test). A water droplet was dispensed on the two surfaces using a syringe pump. A needle was used to dispense the water droplet. The needle was coated with a hydrophobic material to avoid the needle's effect on the contact angle measurements. A side view image was taken and measured.

### Sample preparation and EIS measurement

**Figure 5:**
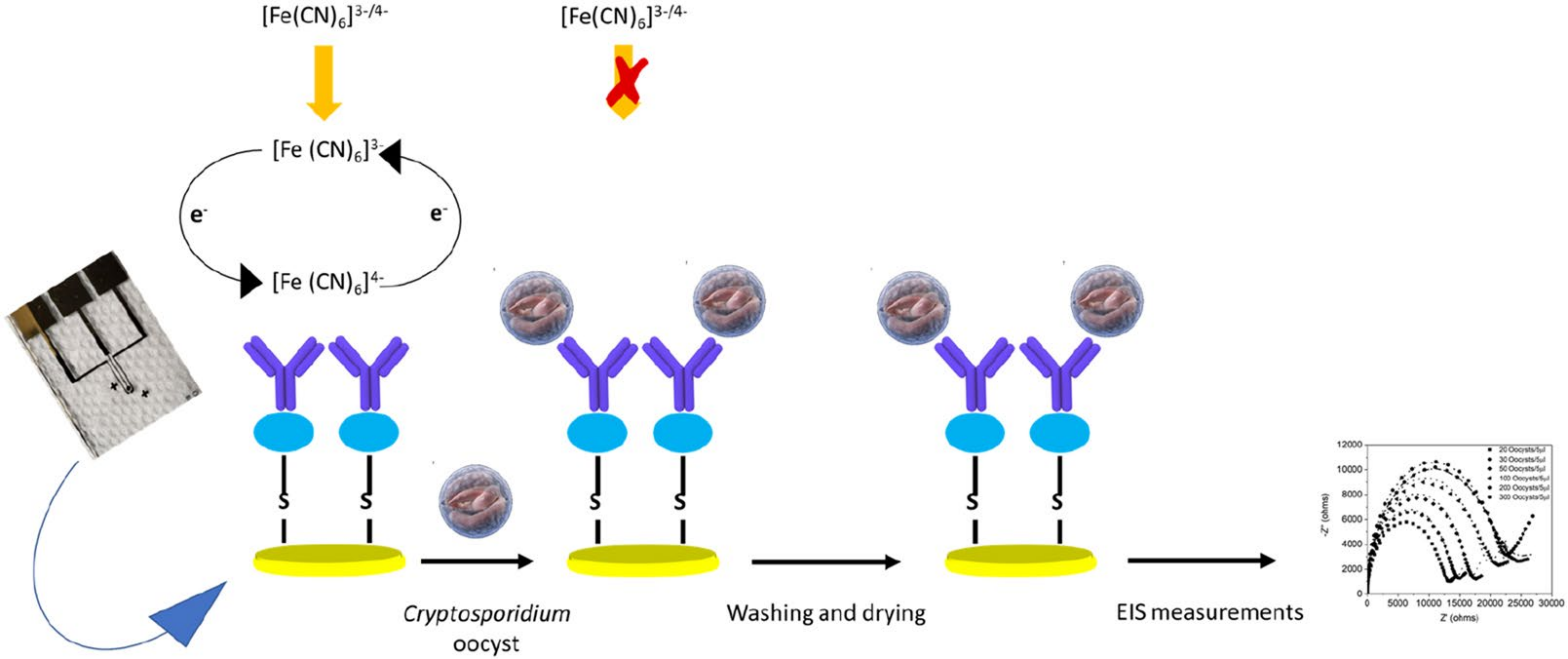
A schematic illustration of sample preparation and electrochemical measurements. In the absence of *Cryptosporidium* oocysts, the [Fe(CN)_6_]^3−/4−^ redox probe permeates the monolayer and reaches the surface of the WE. The redox probe, [Fe(CN)_6_]^3−/4−^, at the surface can be either oxidized or reduced, resulting in a charge transfer (current) to or from the surface. However, the presence of *Cryptosporidium* oocysts in the sample increases the sensor film resistance and electron charge-transfer permittivity, measured by electrochemical impedance spectroscopy.

A series of dilutions of *Cryptosporidium* samples were prepared in the PBS buffer. Typical dilutions were 300, 200, 100, 50, 30, 20, and 0 oocysts in a final volume of 5 µL. The modified SAM/anti-*Cryptosporidium* antibodies microfabricated Au WE were incubated with different *Cryptosporidium* concentrations in a final volume of 5 µL for 20 min at room temperature (23 °C). Subsequently, all electrodes were washed with PBS to remove any loosely bound and unbound *Cryptosporidium* from the WE surface. The samples were blown dry in the air, followed by measurements. The EIS measurements were carried out using the VersaSTAT 4 electrochemical station (Princeton Applied Research) before and after the incubation with different concentrations of *Cryptosporidium* oocysts. All measurements were repeated three times to confirm the detection limit and conduct a statistical error analysis (Fig. 5). The blank electrodes (Au‐thiolated protein/G/BSA) were prepared using the same procedure shown in Fig. 2, excluding the antibody step.

The results were evaluated using the Randles equivalent circuit in the ZView software. The equivalent circuit consisted of the double-layer capacitance at the electrode surface (C_DL_), the solution resistance (R_S_), Warburg impedance (Z_W_) and charge transfer resistance (*R*_CT_). The real (Z′) and imaginary (Z′′) parts of the impedance were plotted in Nyquist diagrams. The charge transfer resistance occurs in the kinetically-controlled high-frequency region; whereas, the Warburg impedance was observed in the diffusion‐controlled low-frequency region^39^. The binding of *Cryptosporidium* oocyst to the modified Au electrode results in increasing *R*_*CT's*_ value, which appears as a larger semicircle in the plot. The changes in the *R*_CT_ between blank and after capturing *Cryptosporidium* were compared. The equation of $\% {\Delta R}_{CT} = {(R}_{CT (\mathrm{with Cryptosporidium})}- {R}_{CT \left(\mathrm{blank}\right)})/{R}_{CT \left(\mathrm{blank}\right)} \times 100$ was used to calculate the relative change of the charge transfer resistance (Δ*R*_*CT*_ (%)) as a function of the *Cryptosporidium* concentration (the six different cell concentrations used here range from 20 to 300 cells/5 µL).

### Calibration curve and limit of detection (LOD)

To assess the applicability of the anti-*Cryptosporidium* antibodies-modified microfabricated sensor for detecting *Cryptosporidium*, the calibration curve was constructed by plotting *R*_*CT*_ versus the number of the *Cryptosporidium* cells. The limit of detection (LOD) was calculated using the following expression: LOD = 3SD_b_/*m,* where SD_b_ is the standard deviation of the blank and *m* is the calibration curve slope^40,41^.

## Results and discussion

### Preparation and optimization of biosensing layer

**Figure 6:**
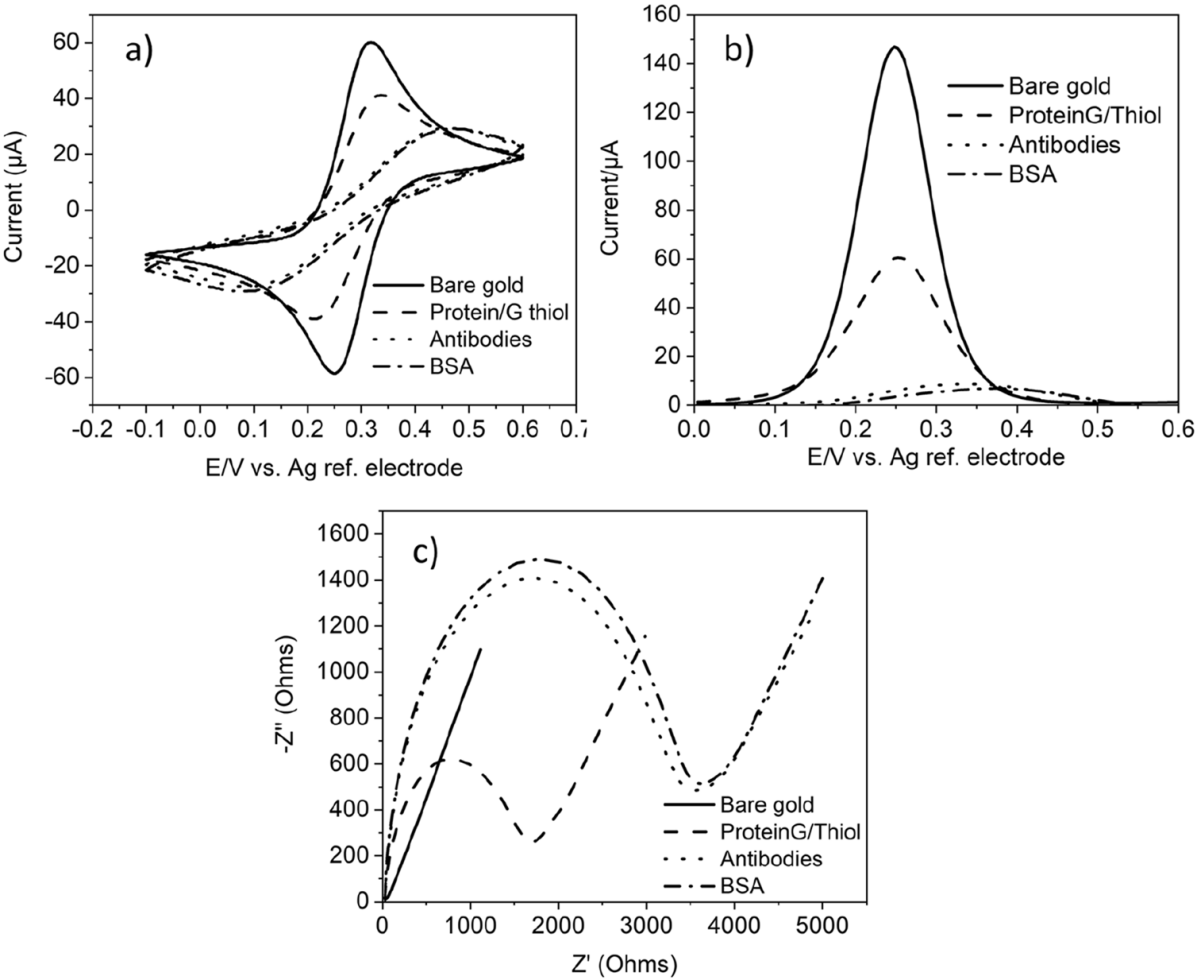
Characterizations of the on-chip label-free immunosensor for the detection of *Cryptosporidium.* (**a**) CV, (**b**) SWV, and (**c**) EIS for the bare Au electrode, added protein/G/thiol, immobilized anti-*Cryptosporidium* antibodies, and added BSA. The measurements were conducted using the microfabricated Au working electrode, microfabricated Au auxiliary electrode and an external Ag/AgCl reference electrode. All electrochemical measurements were started at the OCP and were performed in the presence of an electrolyte which consisted of a 5 mM [Fe(CN)_6_]^3−/4−^ aqueous solution with 1 M NaClO_4_ as the supporting electrolyte. The CV measurements started with a 30 s quiet time followed by a positive scan polarity which swept between the potential range of − 200 mV to + 600 mV (against Ag/AgCl) at a scan rate of 50 mV/s. SWV measurements were conducted in the potential range of − 100 mV to 600 mV with an amplitude of 25 mV and a 2 s quiet time. EIS measurements were performed in the frequency range of 0.1 Hz–100 kHz with a 5 mV amplitude and started with a 30 s quiet time.

The CV, SWV and EIS result in Fig. 6a,b show that there is a decrease in the charge transfer (and hence a decrease in the current and an increase in the impedance) after modifying the electrode surface with protein/G/thiol, after immobilization of antibodies, and after blocking with BSA. The current decrease confirms the formation of SAM, immobilization of the antibodies and the surface blocking step. This is due to the increase in the surface monolayer thickness after each modification step, increasing the separation between the oxidation and reduction peaks of the [Fe(CN)_6_]^3−/4−^. Furthermore, the current decrease confirms an increase in the film resistance and electron charge-transfer permittivity, further confirmed using EIS as shown in Fig. 6c. The data clearly show a significant increase in the *R*_CT_ values form 20.42 ± 0.5 Ω (bare gold) to 3187 ± 3.2 Ω (anti-*Cryptosporidium* antibodies modified surface). The increase in *R*_*CT*_ is due to the formation of an electrically inactive layer (causing the isolation and consequently preventing the charge transfer to the electrode) after each modification step.

**Figure 7:**
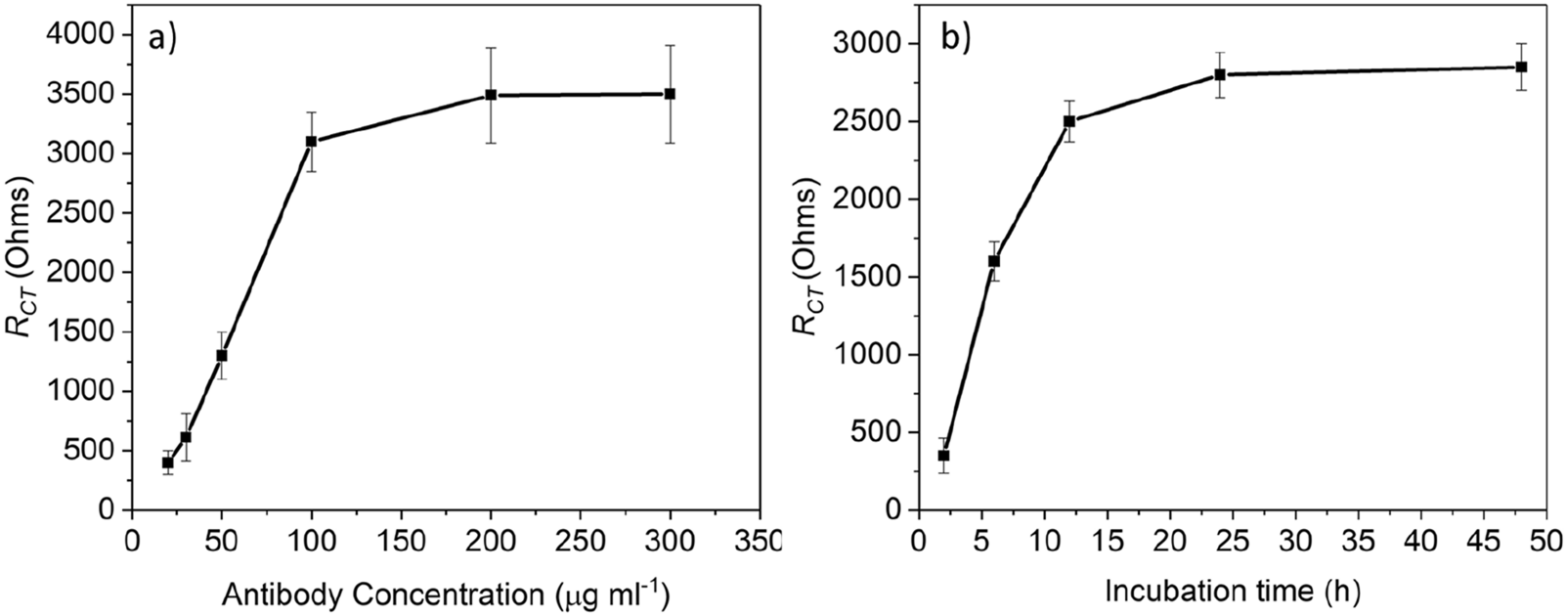
Optimization of the antibody's concentrations and binding time. (**a**) *R*_*CT*_ value for different concentrations of anti-*Cryptosporidium* antibodies, (**b**) *R*_*CT*_ values for the binding time of anti-*Cryptosporidium* antibodies on the sensor surface.

During fabricating and preparing the on-chip immunosensor, the concentration of the biological recognition element (antibodies) and incubation time on the surface was of great importance for optimizing the sensor's analytical performance. Such optimization will increase the amount of the molecular recognition element available on the surface for capturing the *Cryptosporidium* oocysts. For this purpose, different concentrations of antibodies (20, 30, 50, 100, 200 and 300 µg/mL) were immobilized on the sensor surface to determine the optimum concentration required to achieve the highest performance. The ∆*R*_*CT*_ changes in Fig. 7a confirm that immobilization capacity is a function of the dilution: the ∆*R*_*CT*_ was increased to a maximum value at a concentration of 100 µg/mL and then slightly increased with a concentration > 100 µg/mL. The results suggest that the sensor's optimum analytical performance happens using 100 µg/mL of antibodies (chosen here for all experiments).

The incubation time of the molecular recognition element on the surface is also a crucial parameter that influences the impedance measurements (∆*R*_*CT*_) and the sensor's analytical performance. Figure 7b shows the incubation time's influence on increasing the amount of antibodies available for capturing the target pathogen. It is clear that ∆*R*_*CT*_ increases as the antibody's incubation time increases, reaching a plateau after 24 h. Thus, 24 h was used as the optimal incubation time for the antibodies in all subsequent experiments.

### Surface characterization

Contact angle measurements were employed to further confirm the surface's functionalization by SAM and antibodies (see Fig. 4). Contact angle measurements, measuring the advancing and receding contact angles, reveal the surface's level of wettability. In essence, the formation of SAM and the immobilization of antibodies result in a significant increase in the surface's wettability^3,4^. The measured advancing and receding contact angles for the bare gold were 88° and 42° (± 1°), respectively, and 53° and 3° for the SAM-antibodies modified surface, respectively. The decrease in the advancing and receding contact angle values confirms that the modified surface is extremely hydrophilic compared to the bare gold surface. Furthermore, the results confirm the formation of SAM-antibodies film on the Au surface.

### Electrochemical detection of *Cryptosporidium* oocysts

**Figure 8:**
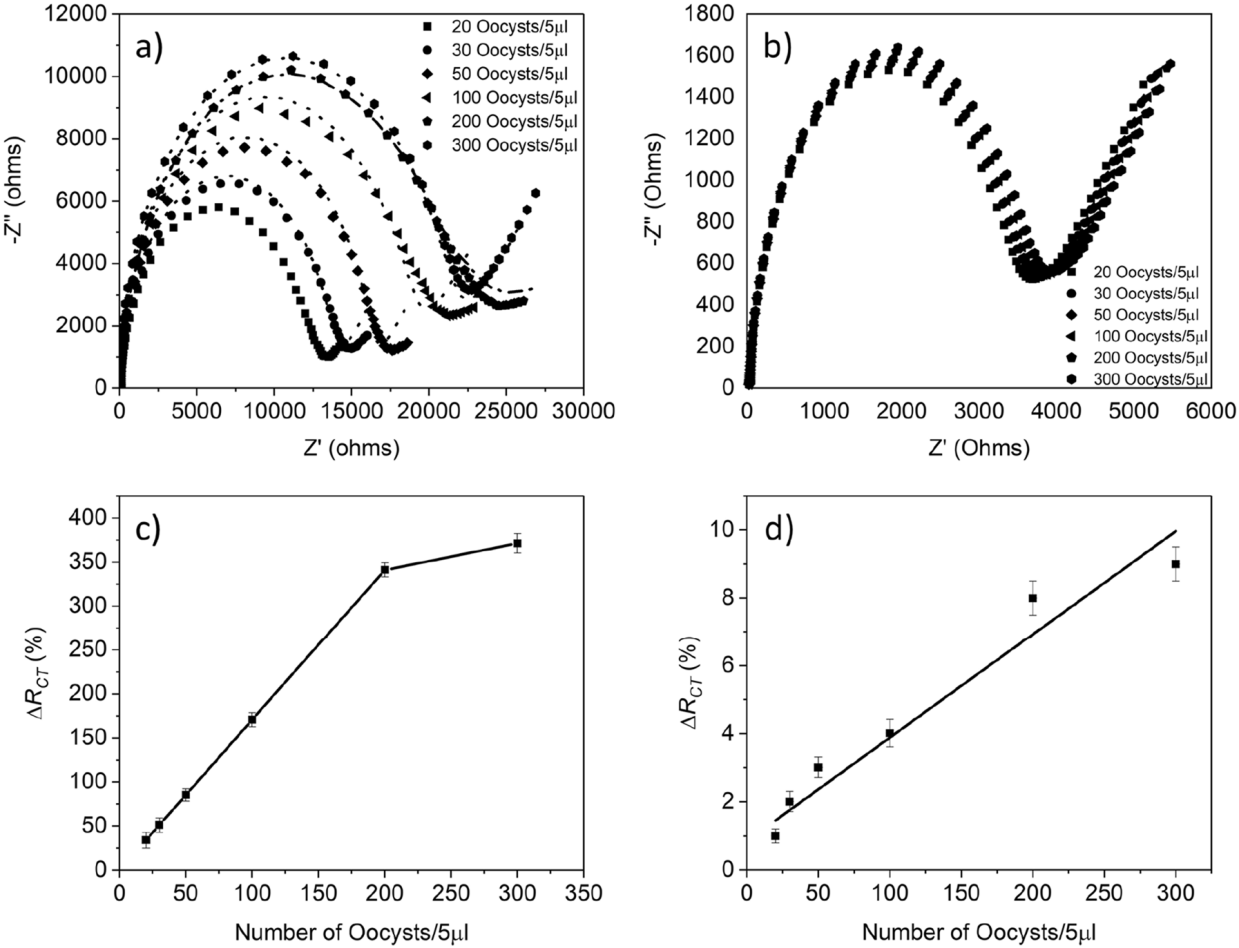
Responses of on-chip-based microfabricated immunosensor to *Cryptosporidium* oocysts. (**a**) and (**c**) EIS measurements and calibration curve for different concentrations of oocysts (20 cells/5 µL, 30 cells/5 µL, 50 cells/5 µL, 100 cells/5 µL, 200 cells/5 µL, and 300 cells/5 µL) in the presence of antibodies, (**b**) and (**d**) EIS measurements and calibration curve in the absence of antibodies for the same number of oocysts.

After confirming surface modification through CV, SWV, and EIS, the interaction of the anti-*Cryptosporidium* antibodies modified sensor with *Cryptosporidium* was studied. The binding of *Cryptosporidium* oocysts was monitored electrochemically in the presence of 5 mM [Fe(CN)_6_]^3−/4−^ (as described in “Sample preparation and EIS measurement”). Briefly, EIS measurements were obtained first for the anti-*Cryptosporidium* antibodies modified microfabricated Au electrode (referred to as blank), and then after incubating the sensor with different concentrations of *Cryptosporidium* oocysts (referred to as test). The measured values are correlated to the number of the *Cryptosporidium* oocysts bound to the modified anti-*Cryptosporidium* antibodies microfabricated Au sensing platform. EIS measurements were acquired for 5 different cell densities (from 20 to 300 cells/5 µL). The real (Z′) and imaginary (Z′′) parts of impedance were plotted in Nyquist diagrams (Fig. 8a) and analyzed by the Randles' equivalent circuit, which best models the EIS spectra using the ZView software. In essence, EIS measures resistance, which increases due to a decrease in the charge transfer and vice versa. Figure 8a shows a dramatic increase in the impedance and decreased charge transfer due to *Cryptosporidium* oocysts' binding to the immobilized anti-*Cryptosporidium* antibodies. Such changes are directly related to the *Cryptosporidium* concentrations; in a very high concentration of *Cryptosporidium* oocysts (> 200 oocysts), the *R*_*CT*_ increase is not as significant as other concentrations. This could be due to the saturation of the sensor. The average values, statistical error, and the lowest detection limit were also calculated. The maximum change in the response due to the presence of different cell densities was observed. The maximum value of the relative change in *R*_CT_ for each number of the captured oocysts was used to generate the calibration curve (Fig. 8c). The curve shows a linear range of up to 200 cells/5 µL and a LOD of approximately 20 cells/5 µL. The results confirm the on-chip microfabricated sensing platform's effectiveness as a label-free, cost-effective and sensitive sensing tool.

To assess the extent of non‐specific binding, a blank sensor was modified similarly to the test sensor (as explained in “Sample preparation and EIS measurement”, excluding the step taken for the antibodies immobilization). The real (Z′) and imaginary (Z′′) parts of impedance were plotted in Nyquist diagrams (Fig. 7b) and analyzed by the Randles' equivalent circuit. Figures 8b,d show a negligible binding of *Cryptosporidium*, e.g., the specific binding of *Cryptosporidium* oocyst in the concentration of 20 cells/5 µL showed a much higher signal than obtained for the same concertation in the absence of anti-*Cryptosporidium* specific antibodies. This proves that the possibility of non-specific binding is not significant. Hence, the developed sensor has a high specificity and selectivity towards *Cryptosporidium*. According to the results of the optimized sensor and calibration curve with error bars (see Fig. 8c), the limit of detection of the sensor for S/N = 3 was found to be 20 cells of *Cryptosporidium* in 5 μL of the sample. Moreover, the maximum relative standard deviation (RSD) of 2.8% at the *Cryptosporidium* concentration of 20 cells/5 μL (4 cells/ μL) and 2.3 at the concentration of 200 cells/5 μL (40 cells/μL) confirmed the excellent reproducibility and repeatability of the developed sensor.

**Figure 9:**
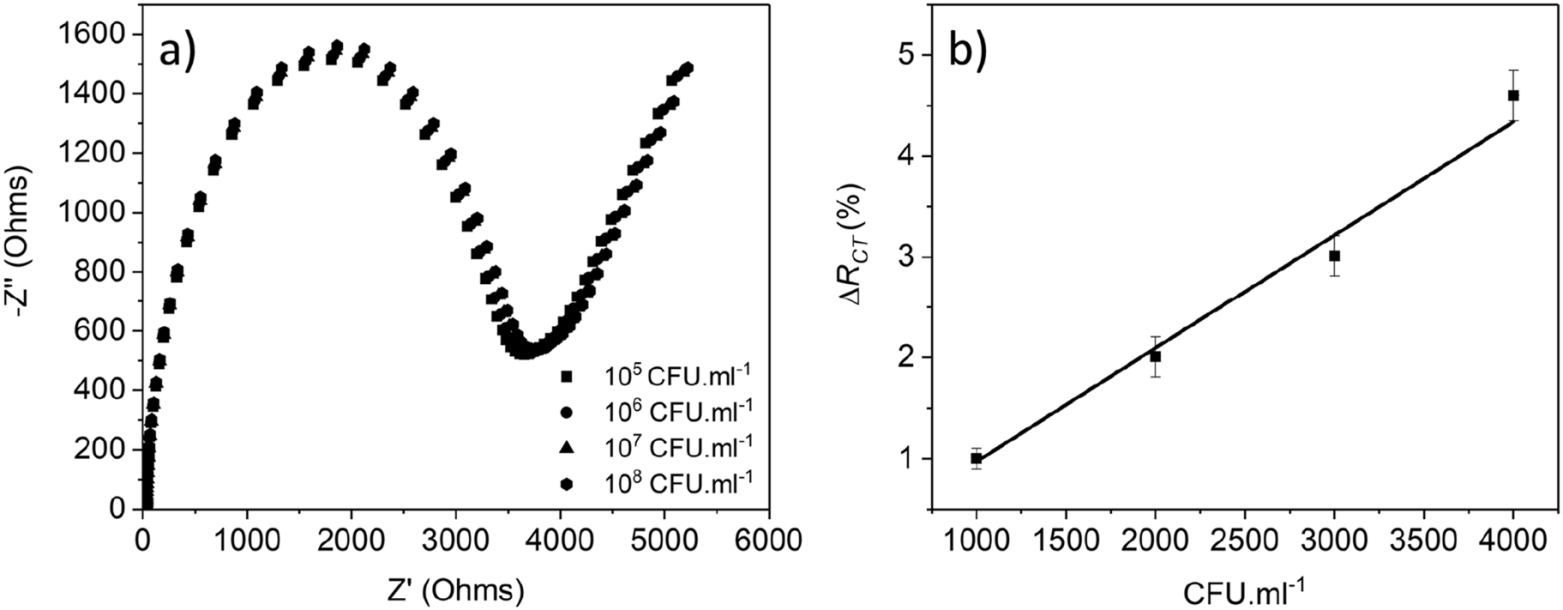
EIS response of the microfabricated immunosensor to the presence of *E. coli.* (**a**) cross-reactivity interaction of four different concentrations of *E. coli.* with the modified sensor, (**b**) calibration curve.

For testing the cross-reactivity, the modified sensor with anti-*Cryptosporidium* antibodies was incubated with four different concentrations of *E. coli* (1000, 2000, 3000, and 4000 CFU/mL)*.* Figure 9a shows a negligible increase in the impedance and the ∆*R*_*CT*_ (Fig. 9b). This increase confirms that there is low interaction with *E. coli* (which could be due to non-specific adsorption) and the sensor's high selectivity towards *Cryptosporidium*. Other non-ionic polymers or blocking agents should be tried to overcome this challenge.

Table 1 shows a comparison of the electrochemical sensors reported in the literature to date for detecting *Cryptosporidium* with the developed sensing platform in terms of detection principle, advantages, and disadvantages.

**Table 1:** Comparative evaluation of the electrochemical sensors previously developed for the detection of *Cryptosporidium* with the sensor developed in this research.

Detection principle	Detection limit	Advantages	Disadvantages	Ref
Square wave voltammetry (SWV—change in current due to the presence of oocysts)	4 cells/µL	Small sample volume (100 µL) High selectivity and sensitivity Low power requirement Robust and easy to miniaturize	Complicated fabrication (use of gold nanoparticles to enhance sensitivity) Use of expensive and non-commercial DNA aptamers	^1^
Differential pulse voltammetry (DPV—change in current due to the presence of oocysts)	0.003 oocysts/µL (3 oocysts/mL)	Short-analysis time High stability Low power requirement	Complicated fabrication (use of gold nanoparticles to enhance sensitivity) Time-consuming Involve many preparation steps Require expensive labels Not suitable for on-site detection	^42^
Amperometry (change in current at a fixed potential due to the presence of oocysts)	0.25 oocyst/µL (1 oocyst/4 µL)	Excellent detection limit (able to detect the amplified mRNA as low as a single oocyst) Low power requirement	Complicated fabrication (use of gold nanoparticles to enhance sensitivity) Involve many preparation steps Require expensive labels A magnet is required for analyte pre-concertation and to capture the bead/liposome complex Time-consuming labelling and preparation steps, including amplification of mRNA before detection Unsuitable for on-site detection and fast-decision-making process	^43^
Electrochemical impedance spectroscopy (EIS—measuring the change of conductivity upon the release of ions from *Cryptosporidium*)	> 10 cells/μL	Label/PCR-free Easy fabrication Small sample volume (110 µL) Cost-effective	Low selectivity Interference with the sample conductivity Generation of inaccurate results A low conductive sample is required for producing reliable and accurate results Sample pre-treatment is required before measurements Time-consuming Unsuitable for on-site detection	^44^
Electrochemical impedance spectroscopy (EIS—measuring the change of the sensor film resistance and electron charge-transfer permittivity due to the formation of the *Cryptosporidium*-antibody complex.)	4 cells/µL	Small sample volume (5 µL) Easy fabrication (no modification with gold nanoparticles to enhance the sensitivity, as reported previously in the literature^1^) Rapid response Cost-effective (> 1 US dollar) Suitable for on-site detection High sensitivity and selectivity Label/PCR-free Low power requirement	Non-specific adsorption. (To overcome this challenge, other non-ionic polymers or blocking agents should be tried)	This work

## Conclusions

A simple, easy to fabricate, and cost-effective label-free on-chip-based EIS-based immunosensor was developed for the sensitive and rapid detection of *Cryptosporidium*. The specific anti-*Cryptosporidium*-antibodies were immobilized on the on-chip-based microfabricated sensor via thiolated protein/G. The EIS measurements were conducted in the presence of the antibodies (test) and the absence of antibodies (blank). The detection limits, sensitivity and selectivity of the sensing platform were obtained. The results showed a linear detection range for *Cryptosporidium* concentrations of 20 cells/5 µL to 200 cells/ µL with a detection limit of 20 cells/5 µL. The specificity and selectivity tests proved the developed sensing platform's high selectivity toward *Cryptosporidium*, demonstrating the developed microfabricated sensor’s applicability for detecting *Cryptosporidium.* Furthermore, a sample volume of 5 µL was used for the measurement using the developed on-chip sensor. This reduces the sample and reagents volume and cost required for a single measurement.

Furthermore, the developed sensor in this research has a great potential to be integrated into a device to capture, separate, and concentrate *Cryptosporidium*. Such a device has been previously developed in the past^45^. This integration will allow for the portable, personalized and POC detection of *Cryptosporidium*. This integration will eliminate the need for trained technicians and specialized laboratories. Moreover, sample treatment by UV or sonication is not needed while using this sensor to detect the whole oocyst.

The developed proof-of-concept of our on-chip electrochemical biosensor is a stepping stone towards creating accessible and ubiquitous pathogen detection methods that could potentially be used in resource-limited settings. Future research efforts are required to test the developed sensor with environmental water samples to confirm the sensor's applicability to replace the fluorescence-based microscopy detection part in the EPA 1623 method. Future studies are also required to test the sensor with other potential interferants and study the capability of the sensor to be extended to detect other biomarkers of interest for other environmental and biomedical applications.
